# COVID-19 and psychogeriatrics: the view from Australia

**DOI:** 10.1017/S1041610220000885

**Published:** 2020-05-12

**Authors:** Nancy A. Pachana, Elizabeth Beattie, Gerard J. Byrne, Henry Brodaty

**Affiliations:** 1 School of Psychology, The University of Queensland, Saint Lucia, QLD, Australia; 2 School of Nursing and Dementia Centre for Research Collaboration, Queensland University of Technology – QUT, Brisbane, QLD, Australia; 3 Academy of Psychiatry, Faculty of Medicine, The University of Queensland, Herston, QLD, Australia; 4 Older Persons’ Mental Health Service, Royal Brisbane & Women’s Hospital, Herston, QLD, Australia; 5 Dementia Centre for Research Collaboration, School of Psychiatry, University of New South Wales, Sydney, NSW, Australia; 6 Centre for Healthy Brain Ageing, School of Psychiatry, University of New South Wales, Sydney, NSW, Australia

“Traveler, there are no paths. Paths are made by walking.”

– from a poem by the Spanish poet Antonio Machado

## Introduction

The continent of Australia is approximately the size of the continental United States, with a population of 25.7 million people, 29% of whom were born overseas (Australian Bureau of Statistics, 2019a). The three largest countries of origin for immigrants are, in order, England, China, and India (Australian Bureau of Statistics, 2019b). The Indigenous population includes both Australian Aboriginal people and Torres Strait Islanders, who represent about 3.3% of the population (Australian Bureau of Statistics, 2018a). These features, as well as Australia’s position as a major modern tourist destination, all play a role in the current global pandemic of Coronavirus Disease 2019 (COVID-19), the disease caused by the novel pathogen severe acute respiratory syndrome coronavirus 2.

Australia recorded its first case of COVID-19 on January 25, 2020 in the state of Victoria, when a man returning from Wuhan, China, tested positive for the virus. The total number of new cases grew exponentially, leveled out at about 360 new cases per day on March 22, 2020, and then fell to its current level of 26 new cases on May 6, 2020, on which date there were 6,849 confirmed cases of COVID-19 in Australia (Australian Government Department of Health, 2020a). Of confirmed cases of COVID-19 in Australia, 96 have died from the virus (Australian Government Department of Health, 2020a). Currently, Australia does not have widespread community transmission of COVID-19; over half a million tests have been conducted nationally (Australian Government Department of Health, 2020a).

From mid-March 2020, Australia has undertaken a series of increasingly stringent measures to limit transmission of the virus. Travel to and transit through Australia is limited to Australian nationals and members of certain Pacific Island nations. Borders between several of the five states and two territories in Australia have been closed. Non-essential businesses (such as restaurants and many retail stores) have been closed, and people have been urged to avoid unnecessary travel and maintain physical distancing when in public spaces.

Australia has a universal national health care system, Medicare, which covers all citizens and permanent residents. Medicare pays for inpatient, ambulatory, and community care provided by public hospitals and subsidizes the cost of inpatient, ambulatory, and community care provided in the private sector by general practitioners (primary care practitioners) and other specialists. It also subsidizes the cost of ambulatory care provided by psychologists, nurse practitioners, and some other health practitioners.

## National mental health access, concerns, and resources

The national “Health Sector Emergency Response Plan for Novel Coronavirus (COVID-19)” was published by the Federal Government on the February 18, 2020 (Australian Government Department of Health, 2020b). The strategic objectives in this plan include characterizing the clinical severity of the disease in Australia, minimizing transmissibility of the virus, minimizing health care system burden, and informing and empowering the public.

Although initially access to mental health services fell under the post-crisis, “stand-down” phase of the national response plan, the federal government (Australian Government Department of Health, 2020c), as well as peak healthcare organizations across the nation, soon realized the need for ongoing and improved access to mental healthcare services. For example, telehealth items have been greatly expanded across all professions, for all Australians irrespective of COVID-19 status, including psychotherapy items for psychologists and psychiatrists, and both telephone and videoconferencing items have been included (Australian Government Department of Health (2020d). Access to flexible ways to deliver “care at a distance” is important with respect to the treatment of older adults, who may have difficulty connecting with mental healthcare providers over videoconferencing platforms and are at increased risk of hospitalization and death from COVID-19. This has not been the case in all countries’ responses to COVID-19, for example, in the United States, the Centers for Medicare and Medicaid Services had stated that services by psychologists count as telehealth only if they include two-way audio and video components (American Psychological Association, 2020a), and this has only very recently been modified to include telephone-only consultations (American Psychological Association, 2020b).

The timing of the coronavirus reaching Australia in many ways could not have been worse. In late 2019 and early 2020, Australia was ravaged by intense bushfires which resulted in great human and wildlife losses, and the devastation of (largely regional) businesses and important national park areas. This national disaster affected all Australian states and territories and followed several years of severe drought that had adversely affected the health and financial well-being particularly of regional and rural farming communities. Although the cumulative fiscal effects of these events – drought, bushfires, COVID-19 – have been written about (*The Guardian*, 2020b), less attention has been paid to the cumulative health and particularly mental health effects of these sequential traumatic events on the Australian public and the difficulties accessing mental health services. Older adults aged 60–69 lost their life in the greatest numbers in Australian bushfires over the last decade; many of these were older persons attempting to save their property, or not evacuating in time (PreventionWeb 2020). Loss of homes, death of partners, lack of mental health support, and (for some) the subsequent trauma of severe flooding following the fires exacted an enormous toll on older adults. Now with COVID-19 restrictions, psychosocial support is for the most part obtained only at a physical distance, over the telephone or other electronic means, and for older persons doubly affected by these disasters and COVID-19, this is a huge strain. Both the recent natural disasters as well as the COVID-19 situation also have severely tested the disaster preparedness of Australia’s nursing home care industry.

Older people in the community, particularly those living alone, who rely on community services such as meals on wheels, home help and community nurses, or access to day centers and communal activities, now find that these are curtailed or ceased with the lockdown conditions. Others decide not to allow services into their home for fear of being infected, with potential deleterious effects on their well-being. Community announcements have been urging neighbors to provide help for older persons by offering to shop, pick up medications, provide food parcels at their door, or simply telephone or leave a note of support. Many older persons may benefit from this outreach, but those at greatest economic disadvantage and socially most isolated may well fall through the cracks.

Older people awaiting elective surgery or having treatments for comorbidities are having to wait, sometimes in pain or to the detriment of their health. Unanticipated risks, such as a greater risk of falls for those waiting for hip replacements, may also lead to premature nursing home admissions. Although elective surgery will be gradually opened back up nationwide in May 2020, because of concerns about their increased vulnerability to the virus, both older adults themselves as well as providers may still be deterred from undergoing such procedures.

The shutdown due to COVID-19 has resulted in major unemployment and an economic crisis that have wreaked havoc globally. Australia has a strong welfare system which has now been boosted by massive government spending in financial support for businesses, the unemployed, rent assistance, and deferred or reduced tax payments. Families have been advised not to visit their older relatives, and some face additional burden in how to provide financial support. Older adults themselves are concerned about their superannuation given the state of world markets, which has also caused much worry and distress for them. These stresses increase the risk of anxiety and depression for older adults as well as younger persons.

## Unique Australian circumstances of COVID-19 transmission

A significant proportion of confirmed cases of COVID-19 acquired the virus, while overseas (64%), persons returning from Europe and the Americas, as well as those returning from cruise ships, make up by far the three largest components of these cases (Australian Government Department of Health, 2020a). In 2018, Australia was the 5th largest cruise market globally, with 1.43 million Australians accounting for approximately 5.2% of all global cruise passengers (from an estimated 30 million each year) (Cruise Lines International Association, 2019; Moriarty *et al.*, 2020). Importantly, a third of the world’s cruising passengers in 2018 were over the age of 60, with 19% aged between 60 and 69 and 14% over 70 (Cruise Lines International Association, 2018).

As the unprecedented COVID-19 pandemic unfolded, cruise ships found themselves denied entry to scheduled ports as infection rates rapidly escalated in popular destinations such as Asia and the Mediterranean. Cruise lines faced no alternative but cancellation and return to home ports for ships mid-cruise. On March 15, 2020, Australia banned cruise ships arriving from foreign ports, but exempted four ships already headed to Australia. One of these, the Australian-based Ruby Princess has since become notorious as the primary carrier of cruise-based COVID-19 into Australia, responsible for 10% of returning traveler cases (*The Guardian*, 2020a) because passengers were allowed to disembark without quarantine. To date, the death toll from COVID-19 attributed to the Ruby Princess has reached 21, and up to 600 more have been infected across Australia (ABC News Australia, 2020). A criminal investigation has begun by New South Wales Police to determine why the ship had been permitted to dock, and passengers to disembark, without quarantine. Subsequent ships allowed into Australian ports have seen all Australian passengers quarantined immediately on disembarkation.

Risk is inherent in all travel, particularly for older passengers and those with chronic health conditions. Travel by older adults with disabilities is on the rise, fueled by an aging population and advances in accessibility and technology. In 2015, more than half of Australians over 65 had some form of disability. Cruise Lines International Association is the world’s largest cruise industry trade association, representing more than 95% of cruise capacity, and has no published guidelines specific to passengers over 65 although many individual cruise lines have internal policies about pre-existing conditions, medications, medical treatments, and mobility aids including guide dogs (Cruise Lines International Association, 2020).

For older travelers, this figure raises safety issues in cruise travel. Medical facilities on board can manage routine medical conditions and the most common shipboard issues, for example, respiratory infections, motion sickness, injuries, and gastrointestinal issues. However, stabilization and evacuation are realities for severe conditions. Medical care is expensive and travel insurance is essential.

Specifically, mobility, cognition, and the stability and management of existing chronic diseases are issues for older cruise travelers. Considerable walking, stability, and agility is required on ships to negotiate compact cabins, use stairs, elevators and gangplanks, and tolerate rougher seas. Wayfinding issues related to cognitive changes can complicate movement about the ship. Older passengers may have to walk more than they typically would at home, become “cabin bound” to avoid walking, or become easily disoriented and reliant on others for help. The capacity to understand and respond to safety procedures such as drills, public address announcements, and notices is critical. Ensuring a longer than adequate supply of all prescription medications, any regular supplements and a first aid kit tailored to individual potential needs is prudent. Remaining active and engaged on the ship and keeping healthy eating, sleeping, and hand hygiene habits all contribute to maintaining wellness.

Older travelers with concerns can consider selecting cruise lines that use sophisticated color-coded digital signage systems for wayfinding onboard, travelling with family or younger companions and taking shorter cruises of 7–14 days. Having effective ways to communicate with family and friends and stay socially connected are a challenge on ships at sea because the Internet is often unreliable and expensive satellite phones are expensive. However, knowing how to use a computer and the Internet, considering using social media, and designating an individual family member or friend to communicate with social networks on behalf of the older person should the need arise can make untoward events more manageable.

During the pandemic uncertainty about what would happen to travelers on board ships, news of the escalation of COVID-19 globally while on board ship with limited communication options, and the lengths of time in strict hotel quarantine affected all passengers, more so older and frailer passengers.

The example of the Ruby Princess cruise ship illustrates the complexity of safely dealing with large numbers of potentially infected people quickly to protect the health of the broader public. The US Centers for Disease Control and the World Health Organization provide general guidance on sea travel and safety precautions for passengers, crew and cruise companies, as do individual countries globally, and do note specific concerns for older people. However, the COVID-19 pandemic and the extensive unplanned at sea and quarantine periods required worldwide highlight the need for a greater level of focus on support of older passengers to ensure that they, their families and cruise lines understand risk. Robust guidelines and procedures relevant to older people must be in place if, and when, cruise travel resumes across the globe, including protocols reflecting scientific and medical knowledge about COVID-19.

## Populations at risk in Australia

### Indigenous persons

Indigenous persons in Australia face heightened risk from COVID-19, due to higher frequency of medical comorbidities than the non-Indigenous Australian population (Broe and Radford, 2018). The National Aboriginal Community Controlled Health Organisation has developed guidelines and resources to assist with management of COVID-19 risk (Australian Government Department of Health, 2020e), with shared decision-making and support of community-based culturally safe practices as foundation. Those considered to be at greatest risk and who should practice enhanced physical distancing include non-Indigenous persons over age 70, medically compromised persons over age 60, and Aboriginal and Torres Strait Islanders over age 50 (Australian Government Department of Health, 2020a), signaling the impact of increased comorbidities at younger ages for this population. Persons living in remote and very remote communities, in terms of both care workers and members of such Indigenous communities, are at particular risk (National Aboriginal Community Controlled Health Organisation, 2020) and have markedly reduced access to hospitals with intensive care unit beds.

There has been a concerted effort to disseminate information and advice about COVID-19 to indigenous communities. Resources including toolkits for healthcare workers and posters and information for communities have been created by Aboriginal healthcare agencies and the Australian Government Department of Health and distributed online. Recordings and videos have been made in Aboriginal languages.

Unfortunately, Aboriginal and Torres Strait Islander prisoners account for just over a quarter (28%) of the total Australian prison population (Australian Bureau of Statistics, 2018b). Given concerns about the spread of the virus in these settings, this remains an area of concern, though as of this time no prisons in Australia have been the focus of COVID-19 cases, whereas nursing homes unfortunately have been a focus of the disease.

### Persons living with dementia, including in nursing homes

As at this writing (6 May 2020), Australia has 63 confirmed cases in nursing home facilities (with 26 deaths) and 31 cases in home care (4 deaths) (Australian Government Department of Health, 2020a). For persons living in nursing homes, special restrictions have been put in place, including restricting visits from persons who have travelled overseas in the last 14 days, or had contact with a person known to have COVID-19 in the past 14 days, people who have symptoms of acute respiratory infection, people who have not been vaccinated against influenza (after May 1, 2020), and children aged 16 and under (Australian Government Department of Health, 2020f). Some facilities have banned all in-person visitors. This level of restriction has been divisive, with some in the community and government condemning the restrictions as too harsh, while the industry itself defends its stance as protective, based on past experiences with norovirus and influenza outbreaks.

Support for worried families is required at this time, particularly as lack of contact with their loved ones continues. Families often provide both help with feeding, ambulation, and socialization; hence, maintaining nutrition and reducing social isolation are issues of increasing importance. Families also often help other residents – some family members will spend much or all day in the facility. Respite services have also been impacted.

Many facilities have provided electronic/digital means for families and residents to connect which has been a boon for residents feeling isolated. Reductions in group sizes and leisure activities, in order to safeguard residents, have been another loss to their routine. Families and residents are being asked to ensure their wishes regarding care and advance care directives are updated. While institutional spread has been mainly contained, there are several facilities where multiple residents and staff have been infected, resulting in several resident deaths in each facility (for the most part in New South Wales, Tasmania, and Victoria). Many residents are fearful and there are reports of increased anxiety and depression.

Other issues are whether to isolate new admissions for 14 days to prevent COVID-19 being introduced to the facility; screening procedures for paid companions or private nurses engaged by families to provide extra care and for external contractors coming into the home; and providing facilities and equipment for barrier nursing or palliative care for infected residents not willing or unable to be hospitalized.

Support for staff in aged care facilities is essential. Staff are affected by reduced family support for residents, concern for their own safety, and worry about transmitting the virus to their own families when their shifts end. Access to and provision of adequate personal and protective equipment (PPE) are challenges for most facilities. Maintaining personal hygiene and physical distancing are difficult with residents who do not have the capacity to understand or remember instructions, placing further stress on staff. Cleaning and catering staff are key to ensuring safety in all facilities. Many health care practitioners are only consulting on residents remotely, possibly limiting access to adequate medical care.

Others who are more vulnerable to infection are people in institutions or group homes such as those with intellectual disabilities and prisoners, especially, if they are older. As culturally and linguistically diverse populations may not access mainstream media, Australian governments have been active in promoting health care and physical distancing messages in multiple languages.

### Older people living with mental disorders

As the availability of informal care is reduced during the COVID-19 pandemic, some older people with mental disorders are running into difficulties. The pandemic has revealed the essential nature of informal support, supervision, and care for these vulnerable older people.

Mental health services for older people are adapting to COVID-19 in several ways. Ambulatory care clinics for existing clinically stable patients are being offered by telephone and videoconference, and many patients are choosing this option (and being assisted if they do not have ready access to appropriate technology). Services are providing clinical case managers with electronic tablets (e.g. iPads) to facilitate video consultations with other members of the multidisciplinary team to ensure good communication around cases.

In most instances, new patients and clinically unstable patients are still being seen face to face, with appropriate pre-consultation inquiries about symptoms and contact with persons with COVID-19, and with physical distancing precautions. Patients are advised to attend at the time of their clinic appointment and not before, to minimize crowding in outpatient waiting rooms. Chairs are spaced and surfaces disinfected regularly. Clinics are looking cleaner than they have for many years.

Telephone and video consultations allow supportive psychotherapy and cognitive behavioral therapy reinforcement sessions to continue, particularly with patients with anxiety, depressive, and psychotic disorders, although some useful clinical information is lost. It is more difficult to conduct consultations with people with more than mild dementia by electronic means, even when a caregiver is present with the patient.

In a recent report, it was estimated that 87% of Australians use the Internet and 69% of the population use social media (Watt, 2019). Even with greater connectivity, there is an emotional toll that comes with lack of human contact; for older adults, this includes loss of contact with grandchildren, and added stress and worry for adult children about aging parents. Australia has historically had well-respected, accessible Internet-based mental health services (e.g. Beyond Blue and The Black Dog Institute). These are particularly important given that regional and remote communities often lack mental health specialists.

### Ethical issues

Older people and their families are fearful that age and/or a diagnosis of dementia will result in them being discriminated against if resources need to be rationed should they require hospitalization, intensive care admission, and ventilation. Families of older persons who are terminally ill with the virus in a hospital or in residential care may be prevented from visiting their older relatives or allowed only one visit to say goodbye and then be obliged to comply with 14-day quarantine in their own home.

Globally, much has been written about increasingly prominent ageist sentiments expressed on social media and even by politicians. Ageism can take many forms, including characterizing older persons uniformly as frail, helpless, and unable to contribute to society (Ayalon *et al.*, 2020). This has led to older people being viewed in some quarters as expendable or, with the usage of the Twitter hashtag #boomer-remover, as being preferentially (and desirably) eliminated by the virus. This not only fuels ageism but also intergenerational conflict, enabling younger people to vent fear and anger at the older generation (Ayalon *et al.*, 2020). An unfortunate effect of such prominent ageist sentiments is that older adults themselves will begin (or continue) to internalize such sentiments, which may have adverse effects on both mental health and positive health behaviors. In reality, in this time of global uncertainly and upheaval, social and intergenerational solidarity are desperately needed (Durant, 2011).

### How institutions and care delivery may change in Australia going forward?

Health care has changed in many countries, including Australia, perhaps permanently. Digital consulting, assessment, management, and prescribing have become the new normal. Practices, regulations, and government and heath fund rebates have been modified accordingly. The community at large has accepted these changes as necessary.

Attention to infection control practices will inevitably continue to be more stringent than before the pandemic, perhaps less so once COVID-19 fades from memory. National plans for dealing with pandemics will be revised and rehearsed. Self-sufficiency in vital utility and health supplies such as medications and PPE will be given higher priority than the short-term economic gain from importing more cheaply from a globalized marketplace.

Research into how to combat viral diseases will be given precedence, and already the Australian government is funding a variety of schemes to find solutions for the present COVID-19 situation, as well as longer term structural and policy-based research on responses to epidemics and their attendant social, economic, and health sequelae. Longer term strategic thinking will be needed. Up until this current crisis, the period of research needed to devise solutions has outlasted the epi- or pandemic, by which time funding dwindles and research (as well as public awareness and government attention) ceases until the next health crisis.

The planet has taken a deep breath. The benefits to the environment from the global shutdown and the likely reduction in deaths from pollution have been widely noted. Whether this will lead to any long-term changes in climate control remains to be seen.

It is becoming clear that in such a global crisis, waiting for the correct course of action to present itself has not worked, and countries and communities must both work together but also forge a path consistent with their unique social values and community norms, being responsive to new developments along the way. This paper began with a quote: *Traveler, there are no paths. Paths are made by walking.* Individuals, as well as nations such as Australia, are walking through this crisis and potentially forging new paths with respect to health care, stewardship of the environment, allocation of resources, and social inequities.

## Final thoughts/summary

Global health crises have occurred periodically over the centuries. The pandemic of COVID-19 is the most devastating worldwide health crisis since the 1918 Spanish Flu. It has forced nations to reconsider their health plans and economic decisions. Gandhi in 1931 stated that “A nation’s greatness is measured by how it treats its weakest members”. We can now be judged by how well we support and care for our older population, the people most vulnerable to COVID-19.
